# Dance Intervention Affects Social Connections and Body Appreciation Among Older Adults in the Long Term Despite COVID-19 Social Isolation: A Mixed Methods Pilot Study

**DOI:** 10.3389/fpsyg.2021.635938

**Published:** 2021-02-25

**Authors:** Pil Hansen, Caitlin Main, Liza Hartling

**Affiliations:** ^1^School of Creative and Performing Arts, University of Calgary, Calgary, AB, Canada; ^2^School and Applied Child Psychology, Werklund School of Education, University of Calgary, Calgary, AB, Canada

**Keywords:** aging, loneliness, body appreciation, dance, improvisation, touch, Kaeja, COVID-19

## Abstract

The ability of dance to address social isolation is argued, but there is a lack of both evidence of such an effect and interventions designed for the purpose. An interdisciplinary research team at University of Calgary partnered with Kaeja d’Dance to pilot test the effects of an intervention designed to facilitate embodied social connections among older adults. Within a mixed methods study design, pre and post behavioral tests and qualitative surveys about experiences of the body and connecting were administered to thirteen participants along with test instruments of loneliness and body appreciation. In the short-term, no significant changes were found on quantitative tests. Exploratory analysis revealed intervention improvements on individual body appreciation questions only. This indication of change was strongly supported by converging qualitative data and identified as relating to: increased connection through task-based collaboration, increased awareness of interpersonal boundaries, and a shift to experiencing the body as responsive. These indications of increased relational capacity were deemed likely to cause further impact in the long term. Examining this possibility and the subsequently arisen factor of COVID-19 risks and restrictions, test instruments were administered again to 10 participants 4 and 5 months after the intervention. A significant increase in loneliness was found. Despite this negative impact of COVID-19 isolation, several positive intervention changes remained detectable and some continued to increase over time. Seventy percent of the participants, who made new social contacts during the intervention and later sought continued contact, improved significantly across all body appreciation measures over the full study. The qualitative data from the last two time-points revealed both consistent values and new, negative changes. While these preliminary findings speak to the durability of intervention changes, they also identify areas of urgent priority to help older adults restore embodied relational capacity that has declined during COVID-19. Within the limitations of a small-sample pilot study, converging mixed methods results support the hypothesis that dance interventions designed for the purpose can positively affect the social inclusion of older adults. Although we recommend further study, these promising results also indicate that dance interventions can help older adults recover from pandemic isolation.

## Introduction

This mixed methods pilot study was conducted in Canada by an interdisciplinary arts-psychology research team at the University of Calgary and in partnership with the established dance company Kaeja d’Dance. The Intervention Phase of the study was completed shortly before COVID-19 broke out and a Phase II assessed the durability of short-term intervention tendencies and effects as well as additional longer-term effects over the first 7 weeks of the pandemic.

The study tested the hypothesis that a dance intervention designed to facilitate meaningful connections through collaborative movement creation tasks, touch, and exchange of embodied memory would have positive effects on the body appreciation and capacity to connect socially of older adults. This hypothesis was both based on the partner organization’s long-term experience working with intergenerational community members and on the theory presented below.

The initiation of the study was motivated by a gap in evidence-based knowledge about the effect on social isolation and loneliness of dance-interventions for older adults, as well as a need to determine and understand characteristics of dance interventions that provide social inclusion benefits.

## Theoretical Framework

### Aging and the Related Issues of Loneliness, Social Isolation, and Body Appreciation

With reference to the growing population of older adults, research attention has recently been directed toward the social and emotional impacts of aging, and how these influence mental and physical health. In the context of the COVID-19 outbreak, the risk of social isolation and loneliness among older adults has become an urgent and prominent concern (Berg-Weger and Morley, 2020; Gorenko et al., 2020; Hwang et al., 2020; Robb et al., 2020; Smith et al., 2020). Gaps in the research result in limited tools to effectively address these concerns.

We know that social isolation and loneliness greatly influence emotional, behavioral, and cognitive functioning (Hawkley and Cacioppo, 2003; Holt-Lunstad et al., 2010; Coyle and Dugan, 2012; Taylor et al., 2018); are strong predictors of mortality (Pantell et al., 2013); and are of critical importance particularly in later life (Hughes et al., 2004). Nevertheless, research demonstrates that at least 10% of older adults frequently feel lonely (Pinquart and Sörensen, 2003). When adults age, their number of social relationships decreases as new relationships become harder to establish, the few connections that remain are deepened (Hughes et al., 2004), and many experience multiple, traumatic losses in their lives (Victor et al., 2000; De Jong-Gierveld and Havens, 2004; Cloutier-Fisher et al., 2011). These changes position older adults at unique risk of developing feelings of loneliness and becoming socially isolated. Thus, loneliness (Jylha, 2004) and social isolation (Jose and Cherayi, 2017) are seen to grow with age.

Although referring to inter-related phenomena, the two concepts used here differ. Social isolation has been defined as a quantitative measurement of the number, type and duration of contact between the individual and their social connections (Wenger et al., 1996; Victor et al., 2000); while loneliness is recognized as a more complex construct reflecting the individual’s subjective evaluation of the level and quality of social contact they participate in contrasted with their desired amount and quality (Weiss, 1973; Andersson, 1998; Perlman and Peplau, 1998).

A study by Smith concluded that loneliness furthermore is an embodied experience because it is “expressed through the participants’ bodies in several ways, including fatigue, tension, withdrawal, and emptiness” (Smith, 2012, p. 45). The limited research available on body satisfaction among older adults does not focus on loneliness and social inclusion. A series of individual findings do, nevertheless, indicate that a multidirectional relationship could exist between body satisfaction, social support, and access to social and embodied activities. Studies have identified negatively perceived physical appearance as a primary source of socially excluding ageism (Hurd Clarke, 2010; Hurd Clarke and Korotchenko, 2011) and interpersonal body acceptance and reappraisal as indicators of positive body satisfaction and well-being (Watt et al., 2017). A positive body image has been linked to older women’s participation in social, public, and active leisure (Liechty and Yarnal, 2010), and people with greater social support have also been found to be more likely to participate in long-term physical activity (Smith et al., 2017). These under-researched connections motivate further study as they seem to affect social isolation and partake in the complex construct of loneliness.

### Arts and Dance Interventions for the Social Inclusion of Older Adults

Helping older adults feel more socially included involves enabling them to pursue lifelong activities, meet their basic needs, maintain important relationships, have meaningful participation in their community’s development, and discover new interests and means to feel fulfilled (Levitas, 2004; Moody and Phinney, 2012; Scharlach and Lehning, 2013). Targeted intervention offers are needed to achieve these desired effects, and arts-based approaches, in particular dance (Keogh et al., 2009), have been recognized as potential methods to provide distinct and relevant benefits for aging populations (Moody and Phinney, 2012; MacLeod et al., 2016; Skingley et al., 2016).

Studies with music, singing, and visual arts interventions have demonstrated positive impacts on older adults’ depression, quality of life, perceived stress, and both mental and physical health (Ronzi et al., 2018). For example, a study conducted by Moody and Phinney (2012) that involved Community-Engaged Arts (CEA) Programming was able to enrich participants’ relationships with each other and with their community by providing an opportunity to connect in new ways, and by strongly emphasizing inter-group collaboration. Participants’ ability to engage with agency in artistic collaboration and public spaces were also identified as important factors (Matarasso, 1997; Moody and Phinney, 2012). Nevertheless, overall intervention effects in this area are still under-researched and inconclusive (Findlay, 2003; Ronzi et al., 2018), in part because the idea of fostering social inclusion through arts-based mediums remains a relatively novel scholarly idea (White and Rentschler, 2005).

Scholars in the fields of dance science and dance psychology have begun researching the impact dance practice can have on older adults’ overall health and wellbeing. However, much of this research has investigated and found physical or cognitive benefits (Keogh et al., 2009; Kattenstroth et al., 2010; Kshtriya et al., 2015; Rodrigues-Krause et al., 2016; Fong Yan et al., 2018; Meng et al., 2019) or focussed on specialized subsets within the aging population (Guzmán-García et al., 2013; Lötzke et al., 2015; McNeely et al., 2015a; Cann, 2016, p. 191–192; Skinner et al., 2018). Yet, it is recognized in the literature that dance-based interventions have great potential to address social isolation and loneliness among older adults (Crumbie et al., 2015), and studies have laid the groundwork for applying dance broadly for this purpose by examining inclusive language and frameworks of delivery (Houston, 2018; Lehikoinen, 2019) and suggesting associated policy development (Cann, 2016).

Dance is noted as being a specifically appealing medium to facilitate social inclusion interventions for older adults because of its many forms, flexible settings, relatively low cost (Keogh et al., 2009), and accessibility (McNeely et al., 2015b). Some scholars theorize that dance may be more enticing to older adults than other options because participants have positive memories of dance from when they were younger (Lima and Vieira, 2007), and because of the supportive, social nature of the artform (McNeely et al., 2015b). Overall, research results appear to support these claims; dance-based interventions have been shown to improve participants’ quality of life, satisfaction with life (McNeely et al., 2015b), mood, and depression (Crumbie et al., 2015; Hyvönen et al., 2020). To these benefits one should add the physical and cognitive effects of dance participation that might support social inclusion. The improved balance and mobility caused by dance practice (Kshtriya et al., 2015) can positively affect the ability to partake in social and cultural offers out of the home. The maintenance of executive functions that dance improvisation has been found to affect (Coubard et al., 2011; Hansen et al., 2020) may support the ability to overcome expectations of social barriers through problem solving and cognitive flexibility.

Previous research has outlined the promising potential of dance-based interventions to reduce the social isolation and loneliness of older adults (Hackney et al., 2007; Duignan et al., 2009; Keogh et al., 2009; Houston and McGill, 2011; Guzmán-García et al., 2013) with reference to the unique format of dance programs and the opportunities they offer for social interaction (McNeely et al., 2015b). Schwender et al. (2018) also found that social dance interventions caused marked improvements in participants’ sense of self and body-awareness of relevance to the connection between loneliness, body values, and bodily practices previously mentioned. It is no surprise then that these socioemotional aspects of dance-based interventions are frequently repeated in the literature (Franco et al., 2015), yet the evidence-base for these claims is both limited and inconsistent (Laban, 2010; Lötzke et al., 2015; McNeely et al., 2015b; Silva et al., 2018).

Although more research is needed into the empirical effect of dance interventions on social inclusion and loneliness, it seems equally important that dance interventions are developed specifically for this purpose. Due to the well-documented physical and cognitive benefits of dance, widespread interventions for older adults tend to be designed with these two benefits in mind. A prominent example of this tendency can be found in the National School of Ballet’s broadly applied programs for older adults in Canada (Skinner et al., 2018). Their promotional videos feature rows of older adults mirroring a younger dance teacher’s movement demonstration without reciprocal interaction. To facilitate social connections, while providing physical and cognitive maintenance that help counter bodily withdrawal, we reasoned that effective dance interventions would require: (1) meaningful tasks of interpersonal exchange with agency to make choices, (2) improvisational exploration of embodied memory, and (3) engagement with proprioceptive and kinaesthetic exercises that may enhance positive body awareness. These principles informed the design of the intervention for our pilot study.

## Study Design

### Overall Design

The triangulation mixed methods model of this intervention study was chosen with reference to the phenomenon studied, the sample size available, and the specific aims of the intervention. Loneliness as it relates to social inclusion is a complex, multifactored construct, which makes it difficult to measure changes over time with accuracy. The partnership opportunity to conduct this pilot study was confined to a small sample of participants, further reducing the efficacy of stand-alone quantitative measures. The dance intervention was developed to support participants’ ability to connect socially through collaborative generation of movement that was based on their autobiographical memory of touch and connection. While standardized quantitative measures of loneliness and body appreciation might produce some relevant information about far transfer trends and effects, the research team expected that qualitative data about relational experiences, comfort levels with touch, and body attitudes would be needed both to triangulate the quantitative results and to consider near and target transfer effects more closely associated with intervention targets in our interpretation.

To distinguish between such effects with clarity, this study draws on Morrison and Chein (2011) to define levels of transfer. Far transfer effects are arrived at using generalizable, standardized measures of changes and are assumed to have impact for participants in situations that differ from the intervention (e.g., when writing an email). Near transfer results are arrived at through measures that are more sensitive to the intervention situation; such results are thus expected to impact the participants in related situations and/or when related factors are consistent (e.g., when involved in an interpersonal activity). The impact of target transfer results is typically limited to similar circumstances (e.g., another collaborative and improvisational dance activity) because instruments primarily measure skills trained within the intervention.

The quantitative and qualitative datasets were processed independently to enable comparative analysis and identification of convergent findings as recommended by Creswell (Creswell and Plano Clark, 2007, p. 62–67). Due to both the known limitations of small sample quantitative data and the more detailed information provided in qualitative data, we anticipated the need to also pursue additional, sequential analysis of the findings, interpreting indications or trends in the quantitative data through the qualitative findings (Morgan, 2017)^1^.

The study was completed in two phases: the Intervention Phase that took place in early December 2019, and a Phase II from early April through May 2020, which replaced a planned follow-up test due to the outbreak of COVID-19. As a result, quantitative test instruments and qualitative surveys were administered at four time points: (1) on the morning of the first intervention day (pre-intervention), (2) in the afternoon of the last and fourth intervention day (post-intervention), (3) 4 months after the intervention (Phase II), which also was 2 weeks after COVID-19 broke out in Canada, and (4) 1 month later and 7 weeks into the COVID-19 outbreak (follow-up). A task-based, behavioral test was only administered at the two time points of the Intervention Phase. An information survey was furthermore distributed pre-intervention and participants were asked to report changes to this information at the Phase-II time point.

The overall study design follows the “Research-based Practice” model for multi-disciplinary research involving the performing arts (Hansen and Barton, 2009; Hansen, 2017). This model enables team members conducting quantitative, qualitative, and artistic research with shared research questions and empirical sources to systematically apply the methodologies of their respective disciplines and to arrive at results with high discipline-specific validity. These results are then exchanged, cross-disciplinarity connections are explored, and application possibilities are identified. Offering additional ethical integrity, the model furthermore ensures that the research team’s arrival at results remains independent from the interests a partner organization may have.

The present study was designed by the research project leader who has expertise in interdisciplinary performing arts psychology (Hansen). Instruments testing participant-reported values were selected in collaboration with the psycho-gerontologist Candace Konnert. The task-based behavioral test was developed collaboratively by the project leader (Hansen) and the partner (Kaeja). The project leader managed recruitment and administered test instruments supported by a research assistant, and the partner delivered the task-based, behavioral test. The partner developed, led, and delivered the dance intervention with dramaturgical and theoretical input from the research project leader. Based on Hansen’s previous research (e.g., Hansen et al., 2014, 2020; Hansen, 2018a, b) and the previously discussed theory, this input focussed on the safety of methods for sourcing dance creation in autobiographical memory, the exchange of such embodied memory, and the prioritization of creation and improvisation over demonstrating and teaching choreographed movement material. Quantitative and qualitative data were processed separately by two research team members with experience in the psychology of aging and education (Hartling) and artistic performance research (Main) who did not witness the intervention. This processing was tested by the project leader who found no errors and strong inter-rater coding consistency (coding differences were small and did not affect the primary patterns found). Preliminary results were exchanged and discussed within the team, further data mining was completed individually to answer questions and review convergences deriving from patterns across the findings, and interpretations (including sequential explanations) were arrived at in team collaboration and while considering the full set of results. Findings, interpretations, and advice for how this knowledge can be used to advance dance offers for older adults were reported to the partner that then began to apply them.

### Participants

Kaeja invited older adults above 60 years in age to sign up for a free dance session on the topic of touch (no dance experience required, first come, first served admission). The advertisement of Kaeja’s invitation was distributed to their news subscriber base and more broadly, through their own social media accounts and the accounts of related community and dance organizations. 26 older adults registered for the dance session.

**FIGURE 1:**
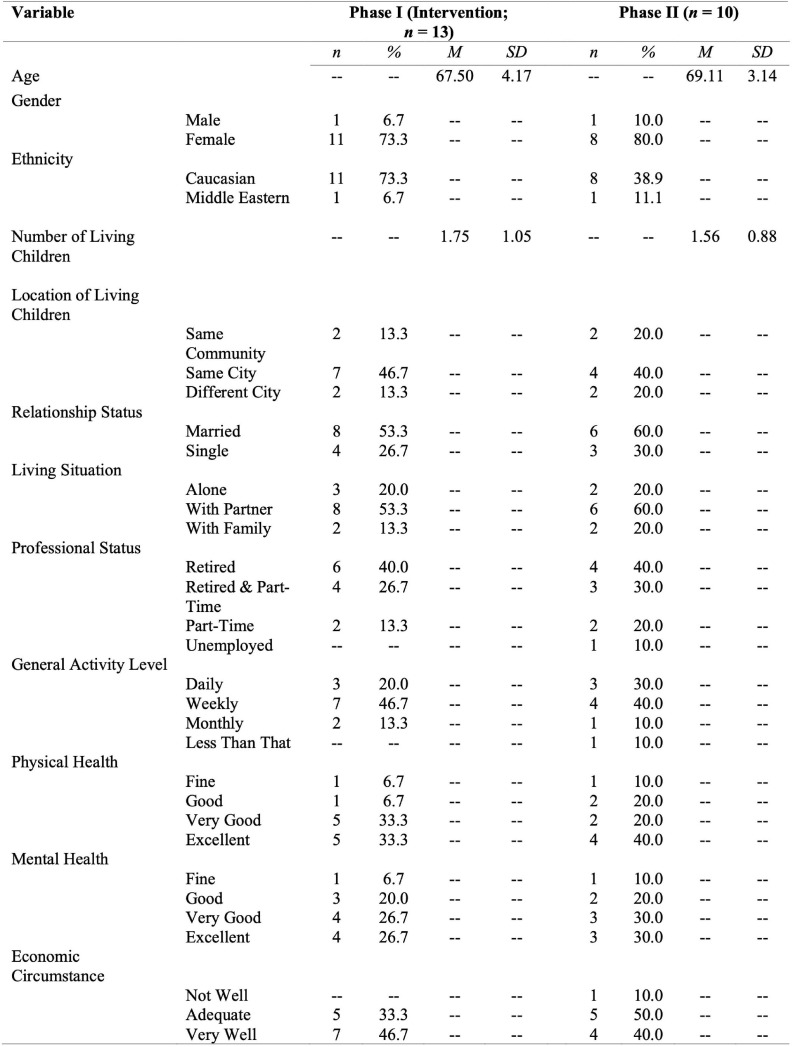
Participant demographic information by group.

These individuals received an independent email from Hansen, explaining that a study had been initiated and inviting them to consider participating on voluntary basis^2^. Thirteen of the 15 participants who signed up for the Intervention Phase made it through a snowstorm that closed public transit on the first intervention day. All 13 study subjects received invitations 4 months later to extend their participation to Phase II, and 10 accepted. Neither of the study phases involved a control group. Consent forms were signed, and a demographic information questionnaire was filled in-person prior to test administration and behavioral tasks engagement in the Intervention Phase. A consent addendum was given on a secure online system (Qualtrics) alongside questions about demographic changes, which then released online versions of the test instruments of Phase II. As evident from the presentation of demographic information in Figure 1, the Phase II group remained comparable to the Intervention group. Note that the specific demographic information collected reflects variables that have been found to impact experiences of loneliness in other studies (Hughes et al., 2004).

### Intervention

The process, regarded for research purposes as an intervention, was a free community dance session, offered over four consecutive days of 3.5 h each. The dance session was titled “The Moving Connections Project” and designed by the mature choreographer Karen Kaeja from our partner organization with research-based feedback from Hansen. For the design, Kaeja drew on more than three decades of creating large-scale dance works with community members, choreographic and improvisational approaches honed through her praxis (see Hansen et al., 2014), and her BA-level training in dance therapy^3^. The work was furthermore informed by—and contributed to—Kaeja’s process of artistic inquiry into intergenerational memory of touch, which would lead to a future professional dance production with community participation. The delivery of the session was supported by Kaeja’s community engagement manager, a rehearsal manager, and four professional dancers. In addition, a psychologist and professional therapist joined the session at the end of each day to witness and offer support for participants. Each day also included a half hour break with snacks to rest and encourage less structured social exchange.

To protect Kaeja’s intellectual property, the creative contents of the intervention are here described in general terms with individual examples, selected to demonstrate the kinds of interaction and experience that were designed to facilitate embodied, social connection^4^.

Each day started with a 10–15 min circle greeting followed by a 20 min warmup. A 45–60 min creation session was facilitated on each side of the mid-session break and every day ended with a circle debrief, inviting participants to share impressions and experiences from the day.

In the circle greeting, each participant was acknowledged and welcomed, a shared space of mutual trust and confidentiality was established, and participants were reminded of how to take care of their bodies during the work (e.g., use a chair or ask a dancer in the room to help adjust a phrase for physical accessibility). The warmup evolved from directing attention toward each person’s proprioceptive experience of their own body while performing gestures through expanding awareness to others in the room while moving in space or interacting with touch. An example of the latter was to take turns “leading” the movement of a partner’s arm by placing one’s wrist under the partner’s and taking part of their weight while moving together.

The first creation session typically involved a process of individual writing in response to a prompt such as “the last time you connected with someone new” or “a meaningful experience of being touched by something or someone.” Participants were instructed to circle three words or phrases and use them to create gestures. Then a scored list of tasks would usually be taped to the wall and participants were asked to work in pairs and later, in groups. They would string their gestures into phrases, teach their phrases to each other, and build choreography with different “conditions.” At times, such conditions were arrived at through improvisation tasks. Participants might for example be asked to cross the space sensing that they were moving through different elements (e.g., water or wind), and then later see the environmental condition listed as a step on a group choreography score. Most scores included options for partners and groups to choose between and required collaborating participants to interpret tasks in addition to engaging with one another’s embodied memories in the form of movement phrases.

The second creation session was largely dedicated to generating one shared group choreography. One by one, over the 4 days, each participant came to the front of the room to share a personal gesture created on the first day. They taught it to the remaining participants and professional dancers while verbally describing the meaning behind the gesture. Everyone in the room was attentive and celebrated the act of sharing. A lead dancer then memorized the gesture, devised a connective transition from the previous gesture to the new one, and worked the full group through a process of learning it step by step. Once 3–4 gestures had been learned individually, the group practiced performing them stringed together. Such smaller phrases of gestures were then combined in larger choreographic sections that were rehearsed, led by the dancer. On the last day, all participants’ gestures had been integrated choreographically and the group performed the full choreography, embodying one another’s memories^5^.

### Quantitative Tests

The two quantitative test instruments administered were the Three-Item version of the UCLA Loneliness scale (3I-UCLA) and the Body Appreciation Scale-2 (BAS-2).

The 20-question Revised UCLA Scale has been a standard measure of participant-reported experiences of loneliness since the 1980s (R-UCLA; Russell et al., 1980). The shorter version of this scale was developed in the 2000s to ease the administration of the instrument (Hughes et al., 2004). It was chosen for this study to avoid the confounding risk of directing participants’ attention toward negative social experiences with a long list of negative concepts. A study testing the psychometric comparability of the two scales from 2004 on 229 older adults found that the correlation between R-UCLA and 3I-UCLA is high (*p* < 001). The questions of the 3I-UCLA scale are: (1) How often do you feel you lack companionship, (2) How often do you feel left out, and (3) How often do you feel isolated from others? Labels included in the response scale are “hardly ever” = 1, “some of the time” = 2, and “often” = 3. The score is calculated as the sum of all items. Higher scores represent higher degrees of loneliness.

The Body Appreciation Scale-2 is a 10 question version of an original 13 question test that aims to turn a common research focus on negative body image and body dissatisfaction to a focus on identifying adaptive body attitudes. This same shift of focus was deemed relevant for our study because of the possibility that adaptive body attitudes might reduce interpersonal barriers. A recent review of BAS-2’s psychometric construct validity found that the scale is strongly related to established measures of body image (Tylka and Wood-Barcalow, 2015, p. 58). The test instrument lists statements such as “I feel that my body has at least some good qualities” or “I appreciate the different and unique characteristics of my body” and asks participants to indicate how frequently they agree with the statements on a 5-item Likert scale ranging from “never” (= 1) to “always” (= 5). The score is calculated as the average of responses to all items. Higher scores represent higher body satisfaction. Subscale scores are reviewed in this study to analyze variance across body appreciation topics and indications of partial changes that relate to qualitative evidence, they do not represent effects in an off themselves.

### Qualitative Data Collection

The qualitative survey administered in the Intervention phase of this study included seven questions, each of which was followed by space for a short, written response. The survey was designed to indirectly reveal changes in behaviors and attitudes toward forming social connections, touch, and the body. The first two questions asked when the participants had last had meaningful, personal exchange with someone they (1) knew well or felt connected to and (2) did not previously know well of feel connected to. These questions instructed the participants to name the context and situation of the exchange and characterize how they felt during the exchange. Question three asked for examples of what made participants feel (non-intimately) connected. Questions four through six inquired about comfort levels around exchange with people participants do not know well that involves (4) sharing personal information, (5) physically touching or being touched, and (6) being emotionally touched. These questions encouraged participants to add examples if an answer depended on the situation. Question seven invited participants to describe their body as they experienced it in that current moment. A research assistant was on site to take dictation from participants who were unable to write; one person made use of this service.

In Phase II, the survey was reduced by removing the two questions about touch (question 5–6). Under COVID-19 restrictions in Western Canada at the time physical touch between non-cohabitants was to be avoided and consequently, these questions were no longer appropriate.

The task-based test of the Intervention Phase was designed to gage whether self-reported changes in attitudes toward social connection and touch manifested in changed behavior. The 40 min test was video recorded and was led by Karen Kaeja. It involved a physical warm-up, private autobiographical writing, individual creation of three gestures from the writing, and either individual or collaborative creation of choreographic phrases by sequencing the gestures. Participants were given the choice between three conditions: (1) working on a solo phrase alone, (2) working on a duet phrase with a partner they did not know well, and (3) working on a duet phrase with a partner they did not know well while also sharing their personal writing. Participants who selected condition 2 or 3 were given the additional option of inserting moments of connection and touch. At the end of this test, participants shared their phrases with the group. The conditions gave participants options, reflecting different levels of interaction and personal sharing with others (which relate to the survey questions about comfort levels with social connection and touch). In addition to observing changes in these explicit choices, the tasks were also designed to provide evidence of more implicit behavioral changes, such as for example the comfort levels revealed by the kinds of touch initiated by participants.

## Results

### Quantitative Results at Far Transfer

#### Methods of Analysis

The objective of exploratory statistical analyses was to identify both significant effects and inconclusive trends as a contribution to the triangulated and sequential mixed methods analyses. All statistical analyses were conducted using SPSS Statistics software. Prior to conducting statistical testing, normality of the data was determined through analyses of mean, median, mode, skewness and kurtosis values; all of which fell within acceptable ranges. Additional visual inspection of the sample was conducted using histograms and boxplots.

Repeated measures ANOVAs were conducted to determine the presence of significant changes in loneliness and body appreciation across the four timepoints outlined: pre-intervention, post-intervention, Phase II (4 months after the intervention), and Phase II follow-up (1 month later). The significance of Mauchly’s Test of Sphericity was analyzed to determine sphericity of the data. In instances where sphericity could not be assumed, the Greenhouse-Geisser correction was implemented to account for such discrepancy.

When main effects from repeated measures ANOVAs were found to be statistically significant, *post-hoc* analyses in the form of paired samples *t*-tests were conducted to determine the specific timepoints where scores differed. Here, despite the presence of multiple comparisons, it was determined that the Bonferroni correction should not be applied to prevent potential Type 1 errors, as doing so to a study with a limited sample size would increase the risk of a Type II error. Additionally, the Bonferroni correction may have masked potential trends in the data, which it would be relevant to further investigate through the qualitative data. Given this decision, all *post-hoc* analyses were planned in advance to theoretically reduce the potential risk of a Type 1 error.

#### Short-Term Intervention Phase Results

Regarding direct, measurable intervention effects between pre- and post-intervention timepoints, there were no significant changes on overall loneliness or body appreciation scores to note.

Exploring subscales for indications of variance across body appreciation themes and partial changes, statistically significant improvement was indicated in responses to two individual body appreciation questions (Q3) “I feel that my body has at least some good qualities” [*t*(11) = −2.35, *p* = 0.039, *d* = 0.67] and (Q10) “I feel like I am beautiful even if I am different from media images of attractive people” [*t*(11) = −2.74, *p* = 0.018, *d* = 0.77).

Hundred percent of the participants furthermore replied on an open participation survey, administered by our research partner, Kaeja, that they would like to participate in dance offers by Kaeja again.

#### Long-Term Findings Discovered in Phase II

From the Phase II survey, administered 4 months after the intervention and 2 weeks into COVID-19 social isolation, it was evident that 80% of the participants acted on their wish to continue dancing with Kaeja and signed up for classes and sessions in the first 4 months after the intervention, although only 40% were able to take in short classes before COVID-19 broke out and the remaining, longer sessions were canceled. Seventy percent of the participants also indicated that they had reached out to connect with other participants from the December dance session that they did not previously know well. In other words, new connections that arose during the intervention were actively pursued afterward.

**FIGURE 2:**
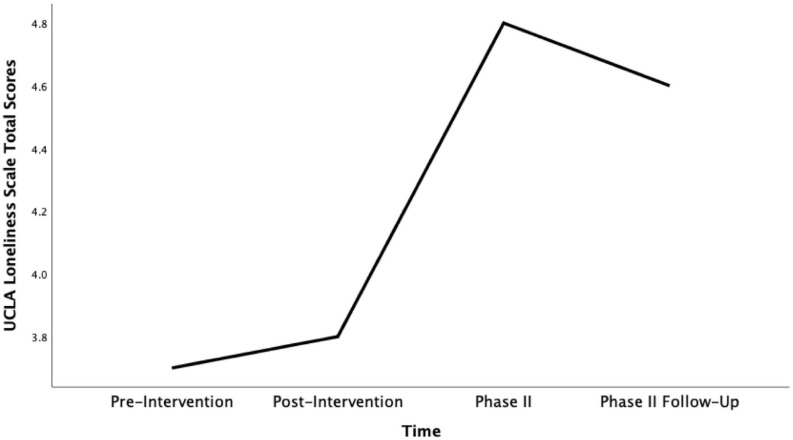
Mean UCLA Loneliness Scale total scores across time (pre-intervention, post-intervention, phase II and phase II follow up).

Taking into account all four timepoints, a significant main effect on loneliness was found [*F*_(__3, 27)_ = 4.52, *p* = 0.011, η*_*p*_*^2^ = 0.33, see Figure 2]. At the Phase II time point (while most still believed COVID-19 measures to be temporary) we found a statistically significant increase in overall loneliness across the full sample of participants from scores reported post-intervention [*t*(9) = −2.54, *p* = 0.032, *d* = 0.80].

**FIGURE 3:**
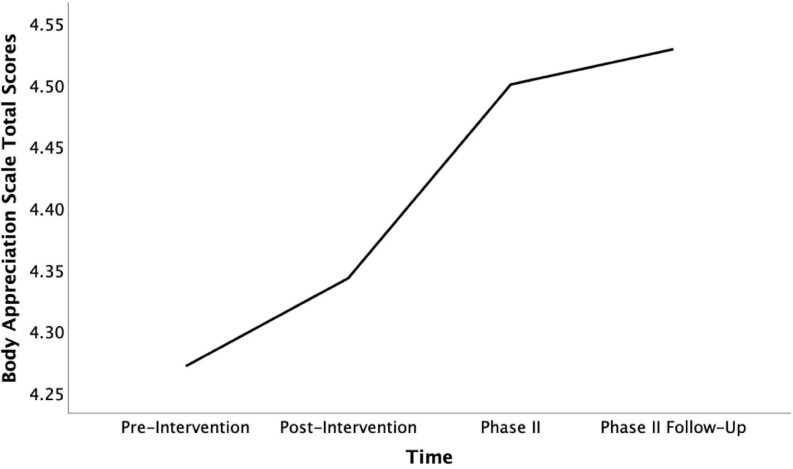
Mean Body Appreciation Scale total scores for participants who continued contact across time (pre-intervention, post-intervention, phase II, and phase II follow-up).

Despite this negative effect, the 70% of the Phase II participants who sought out further contact with individuals from the intervention experienced significant improvement of their overall body satisfaction score across all four points in time measured [*F*_(__3,__18)_ = 3.73, *p* = 0.030, η*_*p*_*^2^ = 0.38]. The increase in scores is particularly pronounced when comparing the pre-intervention timepoint with the follow-up, 7 weeks into COVID-19 social isolation [*t*(6) = −3.42, *p* = 0.014, *d* = 1.31, see Figure 3]. That said, the improvement in their score was gradual and consistent across the four time points, indicating a continued, long-term effect of the intervention on body appreciation levels that is associated with the active and continued pursuit of new social connections made during the intervention.

Note that the ability to sustain or improve body appreciation through COVID-19 measures did not eliminate the increased experience of loneliness. We do not know whether individuals in this demographic would have felt even more lonely without the positive effect of the intervention, but it seems that it is possible to have increasingly positive feelings about the body while also feeling lonely due to isolation or distancing from social connections.

**FIGURE 4:**
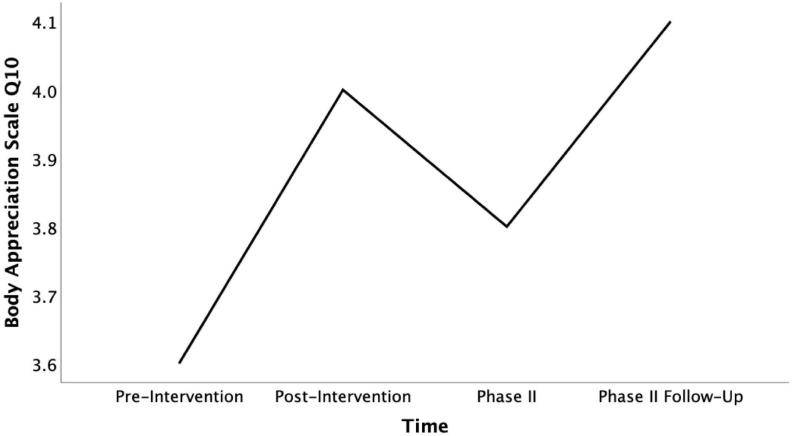
Mean Body Appreciation Scale Q10 scores across time (pre-intervention, post-intervention, pahse II, and phase II follow-up).

**FIGURE 5:**
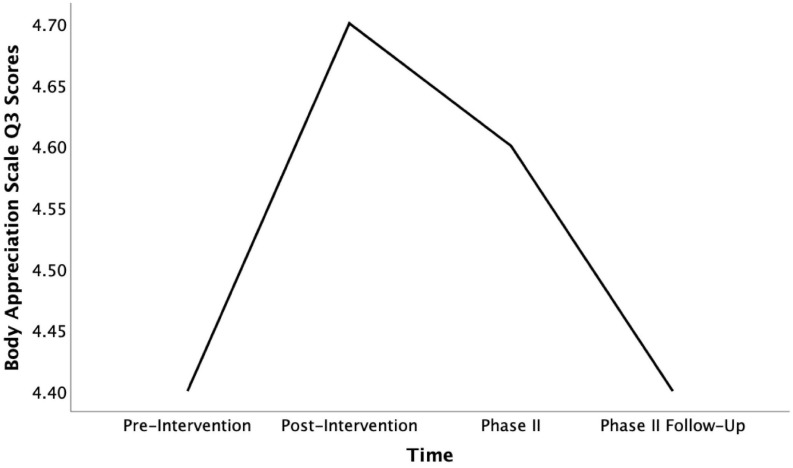
Mean Body Appreciation Scale Q3 scores across time (pre-intervention, post-intervention, pahse II, and phase II follow-up).

**FIGURE 6:**
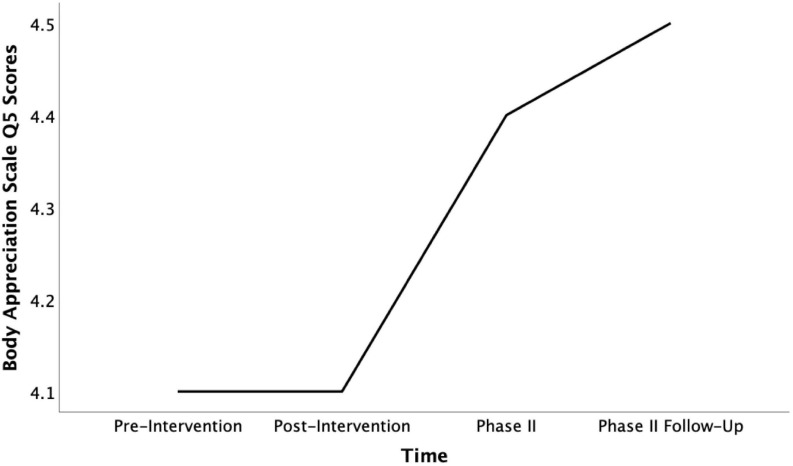
Mean Body Appreciation Scale Q5 scores across time (pre-intervention, post-intervention, pahse II, and phase II follow-up).

Returning to our preliminary exploration of subscore indications for mixed methods purposes, the intervention changes on individual body appreciation questions were still detectable in Phase II. In the case of question 10 about body beauty, scores furthermore significantly increased over the following weeks [*F*_(__3, 27)_ = 3.52, *p* = 0.28, η*_*p*_*^2^ = 0.28]. When comparing scores from the pre-intervention to the Phase II follow-up, this noted increase was even higher than the significant change measured after the intervention [*t*(9) = −3.00, *p* = 0.015, *d* = 0.95, see Figure 4]. Part of the positive intervention change measured on answers to question 3 about positive body qualities remained intact at the time of Phase II. However, the follow-up test revealed that this change declined to the pre-intervention level over longer-term exposure to COVID-19 social distancing measures (see Figure 5). Interestingly, this decline in positive qualities, was more than matched by a significant increase in body appreciation expressed in responses to question 5 “I am attentive to my body’s needs” [*F*_(__3, 27)_ = 3.30, *p* = 0.035, η*_*p*_*^2^ = 0.27], specifically between the pre-intervention and the follow-up timepoints [*t*(9) = −2.45, *p* = 0.037, *d* = 0.78, see Figure 6]. This indicates that participants shifted their focus from positive body qualities to the need to remain physically healthy during COVID-19. In other words, the positive, indicative subscale intervention changes discovered on two questions were still detectable 4 months later, despite the spike in loneliness reported, and the change found on one of these questions continued to grow, while the participants’ body appreciation focus otherwise seemed to shift from qualities to basic needs.

#### Contribution to Mixed Methods Analysis

It is rare that short-term interventions with a small sample yield statistically valid quantitative results on generalizable, far transfer tests. That said, a more granular exploration of subgroup effects and subscale change indications, as the one presented here, can provide preliminary evidence of subgroup impact and indications of possible trends to be further examined through the qualitative data. Within this study, strong convergence was discovered between qualitative results and our findings on the subgroup of participants who sought out continued contact as well as the indicative discoveries from individual body appreciation questions. Together, the quantitative and qualitative results provide promising evidence of the intervention outcomes and the effect on these outcomes of short-term and longer-term COVID-19 measures. We will return to this convergence in the discussion section.

### Qualitative Results at Near and Target Transfer

#### Method of Analysis

The qualitative data was processed inductively using thematic coding. Changes caused over time by the intervention and COVID-19 restrictions were understood through the analytical comparison of coding patterns at the four time points (pre-intervention, post-intervention, Phase II, and follow-up).

Video recordings of the task-based test from the Intervention Phase of the study were first time-stamped and annotated, and then coded in subthemes under the following thematic categories: (1) the participants’ choice of the three conditions for collaboration, (2) methods of collaboration used (e.g., discussion, trying out ideas), (3) evidence of body language (e.g., physical hesitation), and (4) moments of touch (e.g., spontaneous or choreographed). While theme category 1 matched the pre-determined task conditions a priori, categories 2–4 emerged inductively from the empirical data and were then applied systematically to the analysis of the full video dataset. Patterns in coding revealed behavioral changes at target transfer, which were contextualized through the survey findings.

Thematic coding was also used to identify changes (constituting near transfer effects) in the experiences and attitudes that participants self-reported on qualitative surveys at the four time-points of the study. Nvivo research software was used to systematically identify thematic codes and further analyze relationships between thematic coding categories. Participant responses from the pre- and post-intervention surveys were first annotated and coded to synthesize key information with attention to variations. Preliminary thematic categories and subthemes emerged from this work. Participant responses to survey question 1 and 2 about meaningful exchange with social connections produced coding themes about context, type of relationship, nature of exchange, feelings, and self-characterization of the exchange. The thematic coding categories that emerged from answers to question 3 reflected the qualities and methods through which participants felt connected non-intimately. Questions 4 through 6 codes characterized levels of comfort and personal tendencies when forming new connections. Responses to question 7 (“describe your body”) generated body relationship coding themes; in particular, the participants’ attitudes toward their body and focus on specific features/qualities stood out. Comparing preliminary coding patterns, the research team identified more extensive, relational awareness as a qualitative effect of the intervention. To further analyze this pattern with a focus on emergent relationships between the induced themes, the following new thematic categories were applied to the full set of survey data: (1) participants’ attitudes toward the body as state-based or relational/experiential and (2) participants’ awareness of boundaries and reciprocity during exchanges. The described process and codes were subsequently applied to survey responses from Phase II and the follow-up, and results from the two study phases were compared. Relationships between near and target transfer results were furthermore explored.

#### Results of the Behavioral, Task-Based Test

Several participants indicated vocally that their choice between the three conditions (i.e., solo, duet with private writing, and duet with shared writing) was based on interest in trying out different options rather than comfort level with social connection, and thus comparison of these choices did not effectively measure changes. Thematic coding and analysis of the participants’ behavior during their work on the task proved capable of providing evidence. We identified qualitative behavioral tendencies and post-intervention changes, which are reported below with supporting descriptions of observations from the video data.

In the task-based pre- and post-intervention tests, all participants were given 5 min to individually create and sequence three gestures from private autobiographical writing prior to their solo or group work. Pre-intervention results were characterized by a feeling of cautiousness toward the task. This affect was embodied via delayed task initiation and physical expression of hesitation with participants remaining seated for an extended period of time. Once participants began to devise movement, use of space was limited by the overall group formation; participants stood in a semi-circle with a large empty space in the center. Furthermore, the majority of participants worked stationary, creating within their kinesphere and selecting movement isolated to the upper body. Only participant 15, one of the more comfortable dancers, broke from the rigid group formation to explore floorwork.

Behavioral changes in the post-intervention results marked increased comfort with this individual dance creation task. Participants filled the entire studio hall and created individual gestures with an awareness of others’ movements. Faster task initiation allowed for more exploration of space and expanded movement. Gestures utilized the participants’ whole body and different spatial orientations (e.g., more levels, indirect/direct movements, turns). The cautious atmosphere of the pre-intervention test was contrasted by exploration and play in the post-intervention test.

Behavioral changes exhibited in the individual creation processes are comparable to changes in the methods of collaboration participants had access to in the collaborative stage of each test. For participants who selected duets, collaboration in the pre-intervention test was more rigid and structured. Participants used mirroring techniques and verbal explanation to learn each other’s solo. Few suggestions were offered amongst partners and as a result, the solos were sequenced together nearly unchanged. All duets included some degree of mirroring, unison, and stationary, upper-body movement; 4 out of 7 performances had all these features. In contrast, the overall methods of collaboration in post-intervention data were more fluid and responsive. While direct mirroring was still employed to learn choreography, the solos evolved through their translation into duets. For instance, a duet between participant 1 and 4 incorporated a call-and-response technique wherein participant 1 clapped and participant 4 responded by rolling. Such a moment emerged through the collaborative means of verbal negotiation and physical improvisation. Other examples of expanded movement and collaborative possibilities included: the use of cannon timing, explicit accommodation of different physical capabilities, and the exploration of spatial relationships between partners.

Changes in touch also occurred between the task-based pre- and post-intervention tests. While the amount of choreographed touch increased minimally, participants who chose to touch demonstrated changes in comfort levels. Participants who touched during the pre-intervention test typically also described themselves on the survey as generally comfortable with touching or being touched. Some participants who indicated discomfort with physical touch and personal exchange on their pre-intervention survey chose to perform choreographed touch post-intervention. In addition, six participants engaged in instances of “friendly touch,” such as a hug or pat on the back, during the post-intervention test, which is an increase from the two individuals in the pre-intervention test.

In summary, behavioral changes observed were faster task-initiation, more comfortable and expanded use of space, transition from partial- to whole-body movement, more direct (faster) access to collaboration, and new emergence of “friendly” touch. While these changes in part reflected improved dance skills, they also showed improved interpersonal connections and increased bodily comfort of relevance to the study hypothesis and the intervention design objectives.

#### Results of Short-Reply Intervention Phase Surveys

The relevance of the target transfer results was further supported by qualitative findings from both the pre-intervention and post-intervention surveys.

##### Meaningful Connections With New and Old Social Contacts

According to the participants’ self-reported experiences, new connections were formed through meaningful exchange while performing a task and/or sharing an autobiographical memory. Other methods of meaningful exchange included in survey responses were difficult conversations or acts of service—although these methods were more common amongst well-established (old) social connections than newly formed ones. In fact, task-based sharing of autobiography remained the most popular method of exchange across all pre- and post-intervention surveys. This emphasis was equally strong in the pre- and post-intervention tests, and thus not derived uniquely from the intervention. Interestingly, it nevertheless pointed toward several of the characteristics chosen as the focus of the intervention as central for experiences of forming connections in general. Examples of tasks named on the pre-survey included community gardening and serving on an arts board. These examples also included references to light autobiographical exchange. In the post-intervention survey, nine participants explicitly described the Kaeja dance session as a form of meaningful task-based and autobiographical exchange. For example, participant 1 described the dance session as an opportunity to “connect on a richer level with another participant” and later stated, “it’s been wonderful to share stories.” Participant 4, who struggled to verbalize a “painfully personal” autobiographical memory, recognized another participant in a similar situation. Participant 4 described how they used dance to communicate implicitly: “We began to consciously respond in silences to help one another.” Collectively, these examples from the Kaeja workshop illustrated conditions fruitful for the formation of new connections and, in some cases, exhibited moments of emotional touch, which otherwise were only reported from exchanges with well-established long-term connections.

Meaningful exchanges that included “service to” vs. “reciprocity” with a person was another key difference between old and new relationships. Examples of meaningful exchange in old relationships often described a major life event (e.g., serious injury or a graduation) with emotionally loaded language. As a result, these events tended to involve an act of service for another person. For instance, participant 6 supported an injured friend by bringing food and offering company. This participant “felt happy to hear [the friend’s] whole story and let her vent.” On the other hand, new relationships tended to include tasks that were collaborative in nature and involved reciprocal exchange as the foundation for meaningful connection. For participant 1, the conditions for meaningful exchange were created while performing the shared task of cleaning with a future in-law relation. As a result, willingness to engage in deeper conversation was reciprocated, leading to a perceived change in relationship status: “This time we learned so much about each other and our lives. Now we are much closer—a new friend I think.” The importance of reciprocity was further emphasized in responses to the question “What makes you feel connected (non-intimately)?” Here reciprocity was a key quality named alongside common ground, support, and openness.

##### Expressions of Body Value and Experience

When compared to the pre-intervention tests, post-intervention results demonstrated near transfer effects on bodily openness. Two factors related to bodily openness—body awareness and body value—changed after the Intervention Phase. Body awareness was generally characterized using either state-based or relational/experiential language. State-based understandings of the body were expressed as definitions (e.g., “my body is …”), indicating that participants viewed the body as relatively stable/permanent and tended to assign value judgments to certain aspects of it. For example, “I am not happy with my mid-section. I never have been.” On the other hand, relational and/or experiential awareness of the body typically focused more on body feeling as changing according to contexts. Such experiential awareness of the body can be found in participant 9’s description, “I’m currently experiencing my body as healthy and strong… and vulnerable.” Notably, the majority of participants’ body awareness shifted to relational/experiential after the intervention (pre-intervention: 9 state, 2 relational / post-intervention: 3 state, 9 relational). Furthermore, participants experienced changes in body values caused by the intervention from an even split between negative and positive attitudes toward 87.5% majority of positive and neutral (pre-intervention: 7 neg, 7 pos, 1 neu/post-intervention: 2 neg, 11 pos, 3 neu).

##### Comfort Levels With Personal Exchange

Increased awareness of the self in relation to others was found to relate to the increased body awareness described above. Participants’ self-reported comfort levels with personal exchange, physical touch, and emotional touch were typically more contextual in post-intervention surveys (e.g., “The context is very important as it can clarify expectations: why is this personal exchange happening?”), while they predominantly were reflective of personal tendencies in the pre-intervention surveys (e.g., “I am a touchy person”). All participants demonstrated increased awareness of interpersonal boundaries in both the task-based post-intervention test and the post-intervention survey. These changes, however, were particularly useful for participants who were initially uncomfortable with exchange. Participants identified additional factors as crucial in development of new connections. These factors included (1) sustained interaction with another person over time, (2) understanding of social scripts, and (3) clear intentions, and they were described as manifested in Kaeja’s dance session. One participant characterized the session as a “safe” and “ethical” environment, and another participant articulated feeling “understood” by the group during a difficult experience. The movement tasks/exercises were described as “breaking barriers” of physical touch, a way to “empathize, humanize, and connect deeply,” and an opportunity to learn about others and connect with those one might not initially be attracted to. As expressed by participant 14, the dance environment fostered comfort during exchange: “Even if I might think that I might not be comfortable in touching, as soon as the movement begins, barriers are erased.”

##### Intervention Phase Survey Summary

Near transfer results suggest Kaeja’s intervention was an effective method for establishing fruitful conditions for new relationships. Reciprocal collaboration on choreographic tasks and autobiographical exchange produced a greater fluidity of body definition; increased comfort with touch, movement, and space; and expanded awareness of boundaries and how to articulate/respect them. The research team considered it likely that these changes, if maintained, could lead to new relationships and a positive effect on both social inclusion and body appreciation in the longer term, particularly as participants offered examples of meaningful connections made during the intervention in their post-surveys.

#### Results of Short-Reply Phase II and Follow-Up Surveys

##### Meaningful Connections With Social Contacts in Phase II Survey

At the time of Phase II, 4 months after the intervention and 2 weeks into the COVID-19 outbreak, participants showed slight changes in how and why they connected with others. Reports of connections tended to be task-based and less autobiographical in nature. The threshold for what appeared meaningful was lowered, simply “being together” was experienced as a meaningful connection. This indicated a shift in emphasis from development of new connections to basic maintenance of pre-existing ones.

##### Comfort Levels With Personal Exchange and Body Values in Phase II Survey

Collaboration, shared tasks, and awareness of boundaries were predominantly related to COVID-19 risks and comfort levels, pointing toward COVID-19 risks gradually becoming a barrier to many forms of interpersonal exchange. The shifts toward more positive body value terms and toward a more fluid and relational experience of the body that was affected by the intervention remained consistent at this time-point despite the expression of growing concerns about remaining healthy.

##### Follow-Up Survey Consistencies and Changes Across All Topics

Many of these consistencies and developments remained detectable a month later. However, there were a few notable exceptions where longer-term COVID-19 conditions showed an effect on participants. One of these exceptions was a partial decrease in awareness of the body as relational/experiential followed by a matching increase in defining the body as a fixed state. This was mostly the case for the 30% of the participants who did not seek out continued contact with participants in the intervention. The 70% who did initiate such contact were more able to sustain the effect of the intervention on body awareness. The increased focus on physical health on the Phase II surveys did by the time of the follow-up evolve into a focus on fitness and how the body is felt, indicating a shift from concerns to active and positive self-care with relevant body awareness. However, 7 weeks into the COVID-19 outbreak, the respectful, interpersonal understanding of boundaries expressed after the intervention had turned into a hypervigilance and protectiveness of individual bodily boundaries with strong contextual awareness, which likely would make the emergence of new relationships more difficult.

This combination of lasting or recovered intervention effects and new barriers offers strong indications of both the long-term effect of Kaeja’s dance intervention and the new social barriers that COVID-19 risks have caused.

## Discussion

As previously explained, our mixed methods analysis first aimed to triangulate findings through examination of convergence between the quantitative and qualitative results. Secondly, additional explanations of quantitative indications (sub-scale observations) were explored through the qualitative results. The following presentation of these integrated findings includes discussion of the theory and hypothesis of the study.

### Integrated Findings

#### Convergences

Overall, participants’ qualitative descriptions of meaningful connections collected both before and after the intervention featured shared tasks and autobiographical exchange. This strong pattern supports the prior research finding that social relations require deeper connections later in life (Hughes et al., 2004), and further specifies such connections as genuinely personal and based on shared activities. It also validates the part of our intervention design that matches experiences from visual arts interventions (Moody and Phinney, 2012); namely, that social inclusion interventions for older adults benefit from incorporating collaborative tasks. Finally, it supports the value of the second leg of our intervention design reasoning: that basing such tasks on personal memory provides further opportunities to form new connections that feel meaningful. The effectiveness of these approaches is further supported by the converging quantitative fact that 70% of the participants formed social connections during the intervention that they continued to pursue afterward.

Indications of positive changes on body values and individual areas of body appreciation, which were found independently in both the quantitative and qualitative data across all four time points, did—according to the statistical data—evolve over time to a significant quantitative effect across the full test instrument for the 70% of the participants who sought out continued contact with such new social connections. The converging qualitative finding that this same group found it easier to maintain relational body awareness in the long term, despite pandemic social distancing and isolation, supports the association between body appreciation and social connection that was hypothesized in our theory section. In addition, the most consistent body appreciation change across all time points was found in responses to the sub-scale question 10 about appearance and beauty. This, merely indicative, quantitative observation gains relevance when interpreted through the converging qualitative discovery of a consistent, long-term positive intervention effect on value descriptions of the body in the survey responses. Interestingly, question 10 is also the one BAS-2 scale question that most directly addresses the link between negative values of physical appearance, ageism, and social exclusion that has been identified in previous research (Hurd Clarke, 2010; Hurd Clarke and Korotchenko, 2011).

#### Sequential Interpretations

Another discovery, that our results add to these developing understandings of the field, and which was more surprising to us, is the specific importance of body fluidity and relational awareness of interpersonal boundaries. Although body appreciation and kinesthetic awareness would be a beneficial place from which to initiate new social connections, and it might help break—possibly internalized—barriers associated with both ageism and age-related changes in social support structures, the openness and acceptance mentioned by participants as a precondition for forming new connections seem to require additional kinds of body awareness. The experience of the body likely needs to be adaptive and responsive to exchange. This finding offers a focus on more specific qualities than the attention to “adaptive body attitudes” embedded in the quantitative body appreciation test instrument (Tylka and Wood-Barcalow, 2015) or the “proprioceptive and kinesthetic enhancement of body awareness” that formed the third and final leg of our intervention design reasoning.

The examples offered in the qualitative data show us that points of connection with a new individual are best established through reciprocal exchanges, based in shared tasks and a sensitivity to the personal boundaries of the other. Remember that comfort levels with touch remained largely unchanged on the survey and only improved in the behavioral data after the intervention, while the positive experience of negotiating boundaries made a difference for participants who otherwise felt uncomfortable with touch. The same was true for individuals who initially felt less interested in other participants. Their interest grew through collaboration. Looking at Kaeja’s intervention, we do thus have strong qualitative evidence for the effect of tasks to create choreography collaboratively. While proprioceptive and kinaesthetic training exercises likely helped participants gain skills of body awareness of relevance, the collaborative exchange and negotiation was the greatest contributor to the shift toward the adaptive and fluid body experience mentioned above.

It should be noted that the importance of developing and maintaining an adaptive and fluid experience of the body and relational awareness of boundaries through task-based exchanges was further supported by the changes encountered during COVID-19 restrictions. A fluid and adaptive body experience was found easier to maintain during COVID-19 restrictions for participants who continued to have contact with connections made during the intervention.

Despite this positive finding, the loss of access to collaborative exchange during COVID-19 restrictions and the fact of heightened personal risks caused both increased a hypervigilant and non-relational understanding of interpersonal boundaries, which could be connected with the increase in loneliness measured. Meanwhile, both quantitative and qualitative results point toward an increased focus on maintenance of basic physical health, which according to the qualitative data evolved into positive, but non-relational self-care.

#### Concluding on Integrated Findings

It is encouraging to learn that new social connections arose from the intervention and that positive body appreciation changes were sustained and developed into a significant effect over the long-term despite COVID-19. It is useful to now understand how a positive relationship between social connection and body appreciation can be established and maintained through collaboration and relational body awareness.

That said, the increase in loneliness measured and the qualitative changes found give cause for concern. This evidence of negative COVID-19 impact supports researchers of social work and clinical psychology who have been predicting a COVID-19 related surge in social isolation and loneliness among aging populations and recommending the delivery of interventions to address the issue (Berg-Weger and Morley, 2020; Gorenko et al., 2020; Hwang et al., 2020; Smith et al., 2020). Our study adds to these predictions and recommendations the more specific insight that interventions for older adults will be needed to soften self-protective barriers and re-establish the relational capacity that pandemic circumstances have reduced.

Within the limitations of this pilot study, the converging mixed methods results support our hypothesis by providing preliminary evidence that dance interventions designed for the purpose can positively affect the body appreciation and capacity to connect of older adults and thus also their social inclusion. Theoretically, these findings may also partake in the complex factors that cause experiences of loneliness and therefore help reduce loneliness over longer time and under non-pandemic circumstances.

### Limitations

The small sample size underpowered quantitative parts of the study statistically. It made the sample vulnerable to outliers and it inhibited statistical analysis of several relevant demographic factors because some demographically based subgroups became too small for statistical analysis. The invitation to the study was inclusive of all the older adults registered in the dance session. Additional exclusion criteria were not added to eliminate demographic variables. With some exceptions, the sample was drawn from active older adults self-identifying as white and did not reflect the more diverse demographics of most Canadian municipalities. Because the administered tests were measuring changes in attitudes and experiences as indicators of relational capacity, rather than changes in performance, there was no risk of results being confounded by participants learning how to perform better on the tests. The unanticipated pandemic outbreak meant that the long-term intervention effects measured were limited to this specific circumstance. The effects could vary under different long-term circumstances. Addressing both the sample size limitation and the changed follow-up condition, we interpreted quantitative results through the qualitative data to identify and differentiate between causal connections. The fact that only 10 of our 13 participants opted to contribute to Phase II after the COVID-19 outbreak could have affected the comparability of the two phases if the demographics of the groups were different. In our case, the demographics supported comparability.

### Implications

This pilot study provides promising evidence that can help fill the gap in knowledge about the effects of dance interventions for older adults on body appreciation and social inclusion. The study also identifies important connections between these two concepts. Drawing on this evidence, the study points toward intervention characteristics that programs designed for the purpose of addressing social isolation in aging populations can consider implementing to increase the benefits of their offers.

This study furthermore supports growing evidence that such interventions have become even more urgently needed due to the negative effects of COVID-19 restrictions. Older adults will require support to relax protective barriers and regain embodied, relational capacity when COVID-19 restrictions can be safely released. The findings of this study indicate that dance interventions with collaborative tasks and exchanges of memory can offer the needed support.

These implications rest on results that are limited by sample size and homogeneity, and furthermore, are more strongly supported by context-dependent near transfer measures than generalizable far transfer effects. Thus, we recommend the following future steps toward further confirmation and application:

(1) Complete multiple, standardized repetitions of the intervention study with a more diverse sample, that—with a sample size increased through accumulation—might corroborate findings and, possibly, expand their transferability. A comparative analysis will need to factor in changed, post-pandemic circumstances.

(2) Integrate collaborative, task-based creation and memory-based exchange in dance interventions for older adults that are meant to address social isolation.

(3) Offer interventions with the key features identified here for older adults to help re-build post-pandemic social capacity.

## Data Availability Statement

The datasets presented in this article are not readily available because datasets are kept confidential to protect the anonymity of participants as per the ethics protocol of the study. Queries regarding the datasets should be directed to the corresponding author.

## Ethics Statement

The original protocol for the Intervention Phase and a revised protocol for Phase II of this study were approved by the Conjoint Faculties Research Board at the University of Calgary. These protocols included separate informed consent procedures for each study phase.

## Author Contributions

PH completed a detailed outline and wrote the abstract, introduction, study design, and discussion sections, contributed a fourth of the theory section, and revised and integrated the remaining sections. CM wrote the qualitative results section and revised the qualitative data collection section. LH wrote the quantitative results section and three fourths of the theory section, prepared all figures, and revised the quantitative tests section. All authors have reviewed and approved the final manuscript.

## Conflict of Interest

The authors declare that the research was conducted in the absence of any commercial or financial relationships that could be construed as a potential conflict of interest.

## Footnotes

1 Following Morgan’s consolidation of methods for integrating quantitative and qualitative findings, “sequential” here refers to the sequence of analyzing data types that were collected simultaneously (Morgan, 2017).

2 Ethical standards required the study recruitment to take place independently of the dance session advertisement in order to avoid making the dance opportunity dependent on study participation.

3 Lau’s dance therapy program at York University with geriatric care internships at Baycrest hospital (1980–1984).

4 For more information about the intervention, please consult the primary author or future publications on this study.

5 After Phase II was completed and during the pandemic, Kaeja offered a series of online dance sessions in which a large group of community members, including members from the December dance session, learned and rehearsed this group choreography from their own homes—live streamed and together (www.kaeja.org/movingconnections).
